# Systematic overview of Freedom of Information Act requests to the Department of Health and Human Services from 2008 to 2017

**DOI:** 10.1186/s41073-019-0086-2

**Published:** 2019-12-09

**Authors:** Alexander C. Egilman, Joshua D. Wallach, Christopher J. Morten, Peter Lurie, Joseph S. Ross

**Affiliations:** 1 Center for Outcomes Research and Evaluation, Yale-New Haven Health System, New Haven, CT USA; 2 Collaboration for Research Integrity and Transparency, Yale Law School, New Haven, CT USA; 3 Department of Environmental Health Sciences, Yale School of Public Health, New Haven, CT USA; 4 Center for Science in the Public Interest, Washington, DC USA; 5 Section of General Medicine and the National Clinician Scholars Program, Department of Internal Medicine, Yale School of Medicine, New Haven, CT USA; 6 Department of Health Policy and Management, Yale School of Public Health, New Haven, CT USA

**Keywords:** Freedom of Information Act, Department of Health and Human Services, Research transparency

## Abstract

**Background:**

The Freedom of Information Act (FOIA) provides access to unreleased government records that can be used to enhance the transparency and integrity of biomedical research. We characterized FOIA requests to Department of Health and Human Services (HHS) agencies, including request outcomes, processing times, backlogs, and costs.

**Methods:**

Using HHS FOIA annual reports, we extracted data on the number of FOIA requests received and processed by HHS agencies between 2008 and 2017, as well as request outcomes. Processing times were reported in three time increments, < 1–20, 21–60, or 61+ days, and trends in backlog status were also described. Information about costs and fees collected were aggregated.

**Results:**

Between 2008 and 2017, 69.6% of 530,094 HHS FOIA requests were received by the Centers for Medicare and Medicaid Services (CMS), 18.9% by the Food and Drug Administration (FDA), and 11.6% by all other HHS agencies. During this period, CMS processed 374,728 requests, FDA 114,938, and other HHS agencies 61,890. CMS and FDA reduced backlogged requests by 9396 (89.7%) and 4289 (65.3%), respectively, leaving backlogs of 1081 and 2279 requests at the end of 2017. CMS fully or partially granted 60.3% of requests whereas FDA fully or partially granted 72.4%. Of all requests to CMS, 82.0% were considered simple and 18.0% complex; 82.2% of simple requests and 54.9% of complex requests were processed in 20 days, and 5.6% and 29.9% were processed in 61+ days. In contrast, 60.2% of requests to FDA were considered simple and 39.8% complex; 28.8% of simple requests and 9.0% of complex requests were processed in 20 days, and 58.3% and 81.5% were processed in 61+ days. The costs to HHS associated with FOIA requests totaled $446.4 million ($809 per processed request), increasing from $28.1 million ($423 per request) in 2008 to $53.3 million ($1544 per request) in 2017. In total, HHS collected $8.5 million in fees (1.9% of total costs).

**Conclusions:**

FOIA is frequently used to obtain information about HHS and its agencies. With growing costs, minimal fees collected, and lengthy processing times, HHS agencies’ FOIA programs might be made more efficient through greater proactive record disclosure.

## Background

The Freedom of Information Act (FOIA) can enhance transparency and accountability of biomedical research and regulatory agencies by providing access to a wide range of records held by government agencies. The Act requires federal agencies to disclose unreleased records upon request by the public, unless records fall under nine specific exemptions protecting interests such as trade secrets and other confidential commercial information, personal privacy, and national security [1]. At the Department of Health and Human Services (HHS), which has many constituent agencies, such as the Centers for Medicare and Medicaid Services (CMS), the Food and Drug Administration (FDA), the National Institutes of Health (NIH), and the Centers for Disease Control and Prevention (CDC), records subject to FOIA that might be helpful for advancing public health include aggregated billing records, medical product safety and efficacy data, abandoned New Drug Applications, and information related to research integrity [2–4].

Several recent examples demonstrate the beneficial role FOIA can play. Emails obtained by the New York Times through FOIA revealed that alcohol manufacturers had funded and shaped a major study at the National Institute on Alcohol Abuse and Alcoholism (NIAAA) examining the effects of moderate alcohol consumption on cardiovascular disease [3]. In the wake of the Times article, the NIH launched an internal review that found that interactions between NIAAA officials and alcohol industry biased the design of the study to demonstrate a beneficial health effect of moderate alcohol consumption, ultimately leading the NIH to terminate the study and to undertake a wider audit of industry influence on its research activities [5]. In another example, a review of emails obtained under FOIA revealed that the Coca-Cola Company gained access to and influence with employees at the CDC and advanced corporate objectives, such as by downplaying the association between sugar-sweetened beverages and obesity [6]. In these and other examples, information accessed under FOIA has helped uncover undue influence on clinical research as well as on public health practitioners.

FOIA can also be used to provide access to clinical trial data. For example, the Treatment Action Group and the Global Health Justice Partnership used FOIA to obtain clinical trial data including clinical study reports, study protocols, and individual patient-level data, albeit heavily redacted, on the Hepatitis C drugs Sovaldi and Harvoni, enabling the research community to independently assess the safety and efficacy of the drugs [7]. In many cases, FOIA may be the only mechanism for the public to gain access to certain types of trial data of medical products approved by the FDA.

Despite several examples in which FOIA requests have helped inform the clinical and scientific communities, little empirical data exists on FOIA requests to HHS. A previous evaluation of FOIA requests to the FDA focused on classifying requesters and found that of the over 10,000 requests in 2013, 75% were filed by commercial requesters, 12% by news media, and 13% by all other requesters [8]. In addition to the commercialization of FOIA, individuals and news media have raised concerns related to timely processing, improper denials, and costs associated with processing and litigating FOIA requests [9, 10]. Therefore, we characterized FOIA requests to HHS agencies, including request outcomes, processing times, backlogs, and costs, providing a systematic overview of all FOIA requests to HHS from 2008 through 2017.

## Methods

Using HHS’s FOIA annual reports [11], we determined the number of FOIA requests received and processed by HHS agencies between 2008 and 2017. We selected that 10-year span because it comprised the most recent years of publicly available HHS FOIA annual reports at the time of the analysis. We extracted data on request outcomes, based on the agencies’ own categorization: fully granted, partially granted/partially denied, fully denied based on any of the nine FOIA exemptions, and fully denied for other reasons. Based on the distribution of the data, requests were stratified by those to CMS, FDA, and all other HHS divisions (Additional file 1: Table S1).

Agencies are required by statute to respond to FOIA requests within 20 days for any request comporting with agency regulations, unless there are “unusual circumstances,” such as a need to review a voluminous number of records [12]. Requests are assigned to one of two tracks, simple or complex, based on estimated processing needed. The criteria, such as the estimated search and redaction time, used to assign requests to the simple or complex track may vary across agencies. The FOIA annual reports include the number of simple and complex requests processed within 20- and 100-day increments across categories ranging from < 1 to 200 days and 200 to 400 days, respectively. We report processing times in three time increments: < 1–20, 21–60, and 61+ days, based on the 20-day statutory response requirement and because when FOIA requests are litigated and plaintiffs prevail, courts commonly order document production within 60 days [13, 14]. We also collected information about the backlog (requests pending beyond the 20-business-day statutory response period.) Costs, including both processing and litigation costs, as well as fees collected for search time, document review, and duplication were also calculated. All calculations were performed using Microsoft Excel (v.15.28).

## Results

### FOIA request outcomes

Between 2008 and 2017, 69.6% of 530,094 HHS FOIA requests were received by CMS, 18.9% by FDA, and 11.6% by other HHS agencies (Table 1). In this 10-year period, CMS processed 374,728 requests, FDA 114,938, and other HHS agencies 61,890, including some requests submitted before 2008. Over the study period, requests processed by CMS decreased 56.0% while remaining stable for FDA since 2010.

**Table 1:** Outcomes of Freedom of Information Act requests to Department of Health and Human Services Agencies, 2008–2017^a, b^

Year		2008–2009	2010–2011	2012–2013	2014–2015	2016–2017	Total, 2008–2017
CMS	FOIA requests received, No.	76333 (71.4%)^*^	98702 (75.2%)^*^	104788 (77.4%)^*^	52927 (60.6%^)*^	35933 (51.9%)^*^	368683 (69.6%)^*^
	FOIA requests processed, No.	73966 (63.3%)^*^	104993 (75.0%)^*^	105426 (77.3%)^*^	53180 (60.8%)^*^	37163 (52.5%)^*^	374728 (67.9%)^*^
	Fully granted, No.	60059 (81.2%)	58598 (55.8%)	64404 (61.1%)	17428 (32.8%)	8825 (23.7%)	209314 (55.9%)
	Partially granted/ partially denied, No.	118 (0.2%)	1520 (1.4%)	1678 (1.6%)	7294 (13.7%)	5895 (15.9%)	16505 (4.4%)
	Fully denied (exemptions), No.	2925 (4.0%)	13061 (12.4%)	10795 (10.2%)	11164 (21.0%)	11318 (30.5%)	49263 (13.1%)
	Fully denied (non-exemptions), No.	10864 (14.7%)	31784 (30.3%)	28549 (27.1%)	17294 (32.5%)	11125 (29.9%)	99616 (26.6%)
	Backlogged requests, No.	20789^†^	5494^†^	3132^†^	4515^†^	2352^†^	N/A
FDA	FOIA requests received, No.	19769 (18.5%)^*^	18991 (14.5%)^*^	19588 (14.5%)^*^	20182 (23.1%)^*^	21436 (31.0%)^*^	99966 (18.9%)^*^
	FOIA requests processed, No.	32435 (27.7%)^*^	20481 (14.6%)^*^	20194 (14.8%)^*^	20336 (23.2%)^*^	21492 (30.4%)^*^	114938 (20.8%)^*^
	Fully granted, No.	19965 (61.6%)	14754 (72.0%)	14939 (74.0%)	15858 (78.0%)	16448 (76.5%)	81964 (71.3%)
	Partially granted/ partially denied, No.	226 (0.7%)	300 (1.5%)	381 (1.9%)	232 (1.1%)	161 (0.7%)	1300 (1.1%)
	Fully denied (exemptions), No.	190 (0.6%)	175 (0.9%)	353 (1.7%)	367 (1.8%)	335 (1.6%)	1420 (1.2%)
	Fully denied (non-exemptions), No.	12054 (37.2%)	5252 (25.6%)	4521 (22.4%)	3879 (19.1%)	4548 (21.2%)	30254 (26.3%)
	Backlogged requests, No.	11386^†^	8112^†^	4909^†^	4954^†^	4527^†^	N/A
Other HHS agencies	FOIA requests received, No.	10761 (10.1%)^*^	13577 (10.3%)^*^	11007 (8.1%)^*^	14259 (16.3%)^*^	11841 (17.1%)^*^	61445 (11.6%)^*^
	FOIA requests processed, No.	10519 (9.0%)^*^	14565 (10.4%)^*^	10761 (7.9%)^*^	13963 (16.0%)^*^	12082 (17.1%)^*^	61890 (11.2%)^*^
	Fully granted, No.	3673 (34.9%)	4884 (33.5%)	3612 (33.6%)	3815 (27.3%)	3410 (28.2%)	19394 (31.3%)
	Partially granted/partially denied, No.	1591 (15.1%)	2206 (15.1%)	2050 (19.1%)	4124 (29.5%)	3045 (25.2%)	13016 (21.0%)
	Fully denied (exemptions), No.	213 (2.0%)	273 (1.9%)	352 (3.3%)	519 (3.7%)	670 (5.5%)	2027 (3.3%)
	Fully denied (non-exemptions), No.	5042 (47.9%)	7232 (49.7%)	4747 (44.1%)	5505 (39.4%)	4957 (41.0%)	27483 (44.4%)
	Backlogged requests, No.	4656^†^	2475^†^	2798^†^	3471^†^	2185^†^	N/A

*CMS*, Centers for Medicare and Medicaid Services; *HHS*, Department of Health and Human Services; *FDA*, Food and Drug Administration; *FOIA*, Freedom of Information Act

^a^Other HHS agencies in the analysis include the following: Administration on Aging, Administration for Children and Families, Administration for Community Living, Agency for Healthcare Research and Quality, Centers for Disease Control and Prevention, Health Resources and Services Administration, Indian Health Service, National Institutes of Health, Office of the Assistant Secretary for Health, Office of Inspector General, Office of Public Health and Science, Office of the Secretary, and Substance Abuse and Mental Health Services Administration

^b^The number of FOIA requests processed exceeds the number of FOIA requests received because some FOIA requests received prior to 2008 were processed in 2008 or afterwards

*Percentages of the HHS totals; all other percentages are of respective HHS agency totals

^†^Some backlogged requests may be counted twice within each 2-year increment

CMS fully or partially granted 60.3% of requests whereas FDA fully or partially granted 72.4%. The percentage of requests fully or partially granted by CMS decreased by 43.1% while increasing by 20.9% for FDA. CMS and FDA fully denied 99,616 (26.6%) and 30,254 (26.3%) of requests based on reasons other than exemptions, respectively, while denying 49,263 (13.1%) and 1420 (1.2%) based on exemptions, respectively.

No records or withdrawn requests were the most common reasons for full denials (Table 2). CMS most commonly invoked FOIA exemption 6 (personal privacy; 86.1% of its denials related to exemptions) to withhold requested information, while FDA most commonly invoked exemption 4 (trade secrets and other confidential commercial information; 36.1% of its denials related to exemptions) (Table 3).

**Table 2:** Number of full denials based on reasons other than exemptions, 2008–2017

Agency	No records	All records referred to another agency or component	Request withdrawn	Fee-related reason	Records not reasonably described	Improper FOIA request for other reason	Not an agency record	Duplicate request	Other	Total
CMS	48401 (48.6%)	4937 (5.0%)	7096 (7.1%)	5197 (5.2%)	2397 (2.4%)	8677 (8.7%)	2356 (2.4%)	2746 (2.8%)	17809 (17.9%)	99616
FDA	6083 (20.1%)	288 (1.0%)	16644 (55.0%)	1325 (4.4%)	203 (0.7%)	954 (3.2%)	41 (0.1%)	1995 (6.6%)	2721 (9.0%)	30254
Other HHS agencies	6717 (24.4%)	8993 (32.7%)	5111 (18.6%)	449 (1.6%)	656 (2.4%)	1251 (4.6%)	1800 (6.5%)	694 (2.5%)	1812 (6.6%)	27483
HHS total	61201 (38.9%)	14218 (9.0%)	28851 (18.3%)	6971 (4.4%)	3256 (2.1%)	10882 (6.9%)	4197 (2.7%)	5435 (3.5%)	22342 (14.2%)	157353

**Table 3:** Number of times exemptions applied to Freedom of Information Act requests, 2008–2017

Agency	Ex. 1^a^	Ex. 2^b^	Ex. 3^c^	Ex. 4^d^	Ex. 5^e^	Ex. 6^f^	Ex. 7^g^	Ex. 8^h^	Ex. 9^i^	Total
CMS	0 (0.0%)	128 (0.2%)	191 (0.3%)	1440 (2.3%)	451 (0.7%)	54644 (86.1%)	6606 (10.4%)	0 (0.0%)	0 (0.0%)	63460
FDA	0 (0.0%)	23 (0.6%)	420 (11.6%)	1310 (36.1%)	265 (7.3%)	316 (8.7%)	1290 (35.6%)	0 (0.0%)	0 (0.0%)	3624
Other HHS agencies	1 (0.0%)	329 (1.3%)	666 (2.7%)	5111 (20.6%)	3333 (13.4%)	11294 (45.5%)	4063 (16.4%)	0 (0.0%)	0 (0.0%)	24797
HHS total	1 (0.0%)	480 (0.5%)	1277 (1.4%)	7861 (8.6%)	4049 (4.4%)	66254 (72.1%)	11959 (13.0%)	0 (0.0%)	0 (0.0%)	91881

^a^Exemption 1: Classified national defense and foreign relations information

^b^Exemption 2: Information related solely to the internal personnel rules and practices of an agency

^c^Exemption 3: Information that is prohibited from disclosure by another federal law

^d^Exemption 4: Trade secrets or other confidential commercial information

^e^Exemption 5: Inter-agency or intra-agency communications that are protected by legal privileges

^f^Exemption 6: Information that, if disclosed, would invade another individual's personal privacy

^g^Exemption 7: Information compiled for law enforcement purposes that:

7(A). Could reasonably be expected to interfere with enforcement proceedings

7(B). Would deprive a person of a right to a fair trial or an impartial adjudication

7(C). Could reasonably be expected to constitute an unwarranted invasion of personal privacy

7(D). Could reasonably be expected to disclose the identity of a confidential source

7(E). Would disclose techniques and procedures for law enforcement investigations or prosecutions, or would disclose guidelines for law enforcement investigations or prosecutions if such disclosure could reasonably be expected to risk circumvention of the law

7(F). Could reasonably be expected to endanger the life or physical safety of any individual

^h^Exemption 8: Information that concerns the supervision of financial institutions

^i^Exemption 9: Geological information on wells

### Processing times and backlog

**Fig. 1:**
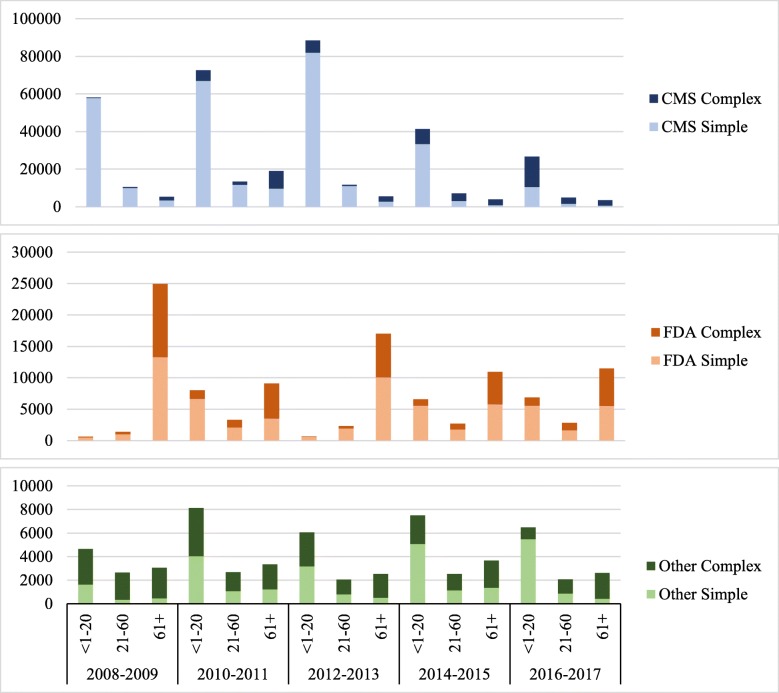
Processing time for perfected Freedom of Information Act requests processed by Department of Health and Human Services Agencies, 2008–2017 (perfected Freedom of Information Act requests are requests for records which reasonably describe such records and are made in accordance with published rules stating the time, place, fees (if any) and procedures to be followed)

Between 2008 and 2017, 82.0% of requests to CMS were considered simple and 18.0% complex; 82.2% of simple requests and 54.9% of complex requests were processed in 20 days, 5.6% and 29.9% in 61+ days (Fig. 1). In contrast, 60.2% of requests to FDA were considered simple and 39.8% complex; 28.8% of simple requests and 9.0% of complex requests were processed in 20 days, 58.3% and 81.5% in 61+ days. CMS and FDA reduced backlogged requests by 9396 (89.7%) and 4289 (65.3%), respectively, compared to 2008, leaving backlogs of 1081 and 2279 requests at the end of 2017.

### Costs

**Fig. 2:**
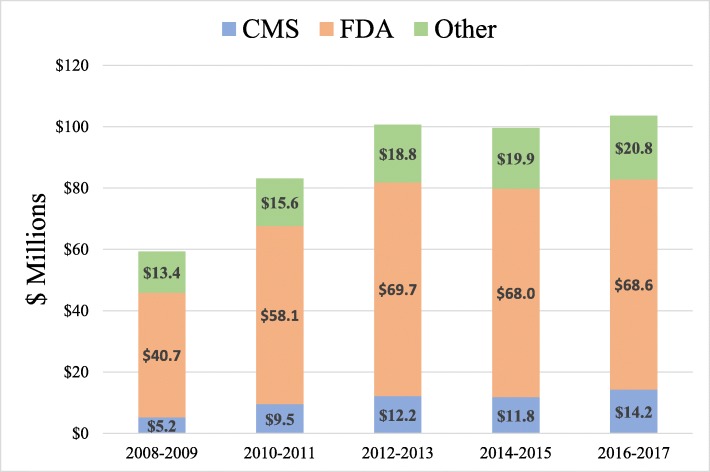
Costs responding to Freedom of Information Act requests by Department of Health and Human Services Agencies, 2008–2017

Over the study period, the costs to HHS associated with FOIA requests totaled $446.4 million ($809 per processed request), including $433.4 million (97.1%) on processing and $13.0 million (2.9%) on litigation (Fig. 2). Total costs increased from $28.1 million ($423 per request) in 2008 to $53.3 million ($1544 per request) in 2017. During the study period, costs to CMS and FDA associated with FOIA requests totaled $52.9 million ($141 per request) and $305.0 million ($2653 per request), respectively. In total, HHS collected $8.5 million in fees (1.9% of total costs).

## Discussion

Between 2008 and 2017, there were approximately 35,000 FOIA requests annually to CMS, 10,000 to FDA, and 6000 to other HHS agencies, demonstrating that FOIA is frequently employed to obtain information about HHS and its agencies. About 40% of FOIA requests to CMS, one-fourth to FDA, and one-half to other HHS agencies were fully denied, most commonly on the basis of no records, requests were withdrawn, or requests were referred to another agency or component. Poorly articulated requests and requests for records that agencies did not collect may contribute to the large number of requests denied on the basis of no records. Many requests are withdrawn after requesters are quoted a processing fee. Agencies invoked exemptions to fully deny requests less frequently, particularly the FDA, which fully denied only 1.2% of FOIA requests based on exemptions. CMS invoked FOIA exemption 6 (personal privacy) for more than three-fourths of the approximately 63,500 exemptions the agency asserted to withhold requested information. However, FDA most commonly invoked exemption 4 (trade secrets and other confidential commercial information). Previously, researchers have suggested that FDA at times invoked exemption 4 even when withholding under that exemption is not warranted under the relevant legal standard [4, 15].

CMS and other HHS agencies processed more than three-fourths and one-half of requests within 20 days, respectively, whereas two-thirds of requests processed by FDA took more than 61 days. Requests to FDA were generally more complex than those to CMS, which likely contributed to FDA’s slower processing time. In addition, FDA addressed potentially complex issues related to FOIA exemption 4 (trade secrets and other confidential commercial information) at a higher rate than CMS, and fully denied requests less frequently, which may have also contributed to FDA’s slower processing speed. However, even within the simple request category, FDA required more than 61 days to process over one-half of requests, compared to 5.6% at CMS and 14.5% at other HHS agencies, suggesting there may be opportunity for FDA to increase its processing speed to more closely align with other HHS agencies. To reduce delays, HHS agencies should consider hiring more personnel and devoting greater resources to responding to FOIA requests. The number of backlogged requests across HHS agencies was reduced by 76.5% over the 10-year span, suggesting that efforts implemented in response to an Obama administration instruction in 2009 to reduce federal agency FOIA backlogs have been effective [16]. The FOIA director at HHS, Michael Marquis, has noted that improved communication with requesters and greater staff accountability were particularly instrumental in meeting the backlog reduction goals [16].

We found that between 2008 and 2017 costs to HHS associated with FOIA requests totaled over $400 million, with requests to FDA comprising nearly 70% of overall HHS costs. The large number of complex requests FDA receives is likely a contributing factor to its disproportionate costs. Costs to HHS per FOIA request more than tripled during the 10-year span. Growth in the number of employees responsible for handling FOIA requests may have contributed to the increase in processing costs. Despite rapidly growing costs, HHS recovered just 1.9% of total costs. A possible explanation for the minimal fees HHS collected is that its agencies are simply not following FOIA fee guidelines, which require commercial use requesters to be charged for any search time, document review, and duplication; news media, educational, or scientific requesters to be charged for duplication only; and all other requesters to be charged for search time and duplication [17]. Another possibility is that fee rates are very low relative to costs. Agencies might consider increasing FOIA fees for commercial use requesters in order to recover more of the agency’s costs. Simultaneously, HHS should strive to make FOIA more affordable and accessible for those requesting information in the public interest, particularly given our finding that nearly 7000 requests to HHS agencies were fully denied for failure or inability to pay the associated fee.

Given growing costs, minimal fees collected, and sometimes lengthy process times, HHS agencies, and particularly the FDA, should consider expanding efforts to proactively disclose records. These efforts might both improve agency transparency and make FOIA programs more efficient. For example, FDA advisory committee materials used to be confidential until they were the subject of FOIA requests, but the FDA changed its practice to proactively release such materials, which has provided useful information for the public and saved the agency time responding to FOIA requests. Notable types of records that HHS agencies might consider proactively disclosing include for the FDA; clinical study reports, final reports of clinical trials that fulfill postmarketing requirements and commitments, and Risk Evaluation and Mitigation Strategies; for CMS, provider level claims and spending data; and for NIH and CDC, expenditures on preclinical and clinical research and development, agency-held intellectual property, and payments to and from industry [18, 19].

## Limitations

This study was limited to aggregate information on FOIA requests to HHS, which did not include information on the types of entities, such as news media, academics, or commercial requesters, who requested records, the information requested and released, and the appropriateness of agencies’ withholding of information. Further, we could not determine whether multiple FOIA requests were for the same materials nor the redundancy in the materials requested, which has implications for HHS’s costs of processing requests.

## Conclusions

Between 2008 and 2017, over 500,000 FOIA requests were made to HHS agencies, nearly 90% of which were to CMS or FDA, demonstrating that FOIA is an important tool for obtaining information about HHS and its agencies. While nearly two-thirds of requests were fully granted and processed within 20 days, one-quarter required 61 days or longer. Requests to FDA were generally more complex, took longer to process, and were costlier to process than those to CMS. Given growing costs, minimal fees collected, and lengthy process times, HHS agency FOIA programs might be made more efficient through greater proactive record disclosure.

## Supplementary information

**Additional file 1: Table S1.** Other Agencies at the Department of Health and Human Services Included in the Analysis.

## Data Availability

The datasets used and/or analyzed during the current study are available from the corresponding author on reasonable request.

## Abbreviations

CDC: Centers for Disease Control and Prevention
CMS: Centers for Medicare and Medicaid Services
FDA: Food and Drug Administration
FOIA: Freedom of Information Act
HHS: Department of Health and Human Services
NIAAA: National Institute on Alcohol Abuse and Alcoholism
NIH: National Institutes of Health
