# Supplementary material for: Systematic overview of Freedom of Information Act requests to the Department of Health and Human Services from 2008 to 2017
Source: Res Integr Peer Rev. 2019 Dec 9;4:26. doi: 10.1186/s41073-019-0086-2 (PMC6900838; doi:10.1186/s41073-019-0086-2)
Supplement: Supplementary file 1 — Additional file 1: Table S1. Other Agencies at the Department of Health and Human Services Included in the Analysis. [file 41073_2019_86_MOESM1_ESM.docx]

**Table S1.** Other Agencies at the Department of Health and Human Services Included in the Analysis.

| Administration on Aging (AOA) |
| --- |
| Administration for Children and Families (ACF) |
| Administration for Community Living (ACL) |
| Agency for Healthcare Research and Quality (AHRQ) |
| Centers for Disease Control and Prevention (CDC) |
| Health Resources and Services Administration (HRSA) |
| Indian Health Service (IHS) |
| National Institutes of Health (NIH) |
| Office of the Assistant Secretary for Health (OASH) |
| Office of Inspector General (OIG) |
| Office of Public Health and Science (OPHS) |
| Office of the Secretary (OS) |
| Substance Abuse and Mental Health Services Administration (SAMHSA) |
